# A Hybrid Approach of Gene Sets and Single Genes for the Prediction of Survival Risks with Gene Expression Data

**DOI:** 10.1371/journal.pone.0122103

**Published:** 2015-05-01

**Authors:** Junhee Seok, Ronald W. Davis, Wenzhong Xiao

**Affiliations:** 1 School of Electrical Engineering, Korea University, Seoul 136-713, Republic of Korea; 2 Stanford Genome Technology Center, Palo Alto, California, United States of America; 3 Massachusetts General Hospital and Shriners Hospital for Children, Boston, Massachusetts, United States of America; Queen Mary Hospital, HONG KONG

## Abstract

Accumulated biological knowledge is often encoded as gene sets, collections of genes associated with similar biological functions or pathways. The use of gene sets in the analyses of high-throughput gene expression data has been intensively studied and applied in clinical research. However, the main interest remains in finding modules of biological knowledge, or corresponding gene sets, significantly associated with disease conditions. Risk prediction from censored survival times using gene sets hasn’t been well studied. In this work, we propose a hybrid method that uses both single gene and gene set information together to predict patient survival risks from gene expression profiles. In the proposed method, gene sets provide context-level information that is poorly reflected by single genes. Complementarily, single genes help to supplement incomplete information of gene sets due to our imperfect biomedical knowledge. Through the tests over multiple data sets of cancer and trauma injury, the proposed method showed robust and improved performance compared with the conventional approaches with only single genes or gene sets solely. Additionally, we examined the prediction result in the trauma injury data, and showed that the modules of biological knowledge used in the prediction by the proposed method were highly interpretable in biology. A wide range of survival prediction problems in clinical genomics is expected to benefit from the use of biological knowledge.

## Introduction

High-throughput gene expression profiling technology has been applied in the studies of many important human diseases [1–5]. Computational methods have also been developed for the analysis of expression data for statistical inference of significant genes [6], classification of disease subtypes [7], prediction of patient outcomes [8], and data mining for biological knowledge [9].

For the prediction of patient outcomes, many existing algorithms focus on the identification of expression signatures of individual genes. These algorithms often first identify genes whose expression indices are significantly correlated with patient outcomes, and then include them individually as independent features in the subsequent feature selection step to build a predictor for clinical outcomes [8]. Despite some successes, this single gene approach has limitations [10,11]. First, although each human disease causes significant disturbance on important biological functions and pathways, profiling of gene expression does not directly measure the activities of these functions and pathways. As a result, in a single gene approach the change of each individual gene might not be significant enough to be selected as a feature in the predictor. Second, measurements on single genes are prone to noises and artifacts from the study design or data acquisition. However, these random noises and artifacts would not be enriched with specific biological functions related to the disease.

To address these limitations, approaches have been developed to analyze gene expression data together with the accumulated prior knowledge in biology and medicine [10–12]. The knowledge on gene functions can be encoded as gene sets, where sets of genes are grouped together by biological contexts such as signaling pathways, chromosomal positions, and concerted responses to various stimuli [10]. A gene set therefore provides the representation of a biological context. Testing on gene sets instead of individual genes in the analysis of gene expression data reduces the dimension of the data in a knowledge-driven way [13].

Several issues need to be addressed when applying the gene set approach to the prediction of patient outcomes. The first is how to incorporate the information of gene sets in a well-established conventional prediction framework, which typically includes the calculation of feature scores, selection of features, summarization of the scores of the selected features into a predictor, and prediction of the survival outcomes of test samples. For example, to calculate the feature score of a gene set, the statistics of individual genes of its members can be summarized for the gene set [14]; alternatively, the expression indices of these individual genes can be summarized directly as the feature for the gene set. The second is how to choose an optimal collection of gene sets in advance. There are numerous collections of available gene sets that reflect different categories of biological knowledge. For example, the molecular signature database (MSigDB) has several gene set collections reflecting chromosome positions, biological functions, regulatory motifs, and cancer modules [10]. Since different gene set collections are derived from different biological context, their prediction power is expected to be different depending on disease problems. Thus, choosing an appropriate collection of gene sets becomes a challenge. The third issue is how to cope with the incomplete existing knowledge in biology and medicine. That is, existing gene sets are based on our current understanding of biology and medicine, which is far from complete. For instance, if a gene set representing a signaling pathway misses a number of downstream genes whose expression levels are regulated by the activity of the pathway, the expression changes of these genes will not contribute to the activity of this pathway even though there are strong signals in the data.

While most of the gene set approaches have been applied to the inference of significant biological functions and pathways, there have been efforts on the prediction problem as well as on the systematic evaluation of the performance [15,16]. However, these studies focus on the classification problem, e.g. prediction of dichotomous outcomes, instead of the regression problem that predicts continuous outcomes such as survival risks. For example, Abraham *et al*. [15] predicted if the survival of a breast cancer patient was less than five years or not, by using classification algorithms such as support vector machine instead of directly predicting the survival risks through regression. Censored samples before five years were removed in their analysis because censored samples cannot be handled in the classification setting.

To our knowledge, there has not been a systematic study of the performance of applying gene set approaches to the regression problem of survival risks. Classification and regression problems are two major branches of prediction analyses. Distinct from the classification problem that predicts discrete outcomes, the regression problem predicts continuous outcomes such as survival risks from censored data. Predicting the survival risks of patients based on genomic data has been widely considered as an important problem in clinical genomics [8,17–19]. To measure the performance of a regression model in the prediction of continuous survival risks, log-rank p-values [20], log-likelihood scores [21] and Harrell’s C indices [22] are commonly used [8,17–19]. In contrast, the classification problems utilize area under curve (AUC), true positive rates, and false positive rates to measure the accuracy of predicting the correct classes.

Moreover, previous gene set prediction methods mostly focused on investigating various summarizations of single gene signatures into a gene set signature to improve the prediction power. For example, Abraham *et al*. [15] tested mean, median, medoid, t-statistics, and principal component analysis summarization methods, and Holec *et al*. [16] tested mean and singular vector decomposition summarization methods combined with various feature selection algorithms. However, there haven’t been much consideration for the optimal collection of gene sets and incompleteness of biological knowledge in gene sets.

In this work, we propose a hybrid method using the information of both gene sets and single genes to predict patient survival risks. The incomplete knowledge of gene sets can be compensated by single genes that are measured genome-widely. Additionally, single genes can partially fill missing information of gene sets due to the non-optimal selection of gene set collections. Gene sets can provide context-level information that single genes hardly capture. Single genes and gene sets are expected to complement each other. The proposed method incorporates the information of gene sets by summarizing single gene expression to gene set expression. It also uses an integrated ‘super-collection’ of gene sets as a sub-optimal gene set collection, and partially compensates the incomplete knowledge of gene sets by including single genes in feature selection. Different from previous methods, the proposed method predicts survival risks directly through the regression of censored data with a Cox proportional hazard model [8]. The performance of the method was evaluated over multiple data sets from patients of trauma and cancers. The result implies the usefulness of the proposed method.

## Results and Discussion

### Robustness of gene set features vs. single gene features

Features of gene sets summarized from its member genes are expected to provide more robust information than features of single genes. Since a single gene feature is based on a single measurement of its gene expression, it can be easily perturbed by experimental noises or heterogeneity of the clinical samples. In contrast, a gene set feature is summarized from measurements on many member genes of the set, which is expected to be more robust to noises and outliers.

**Fig 1:**
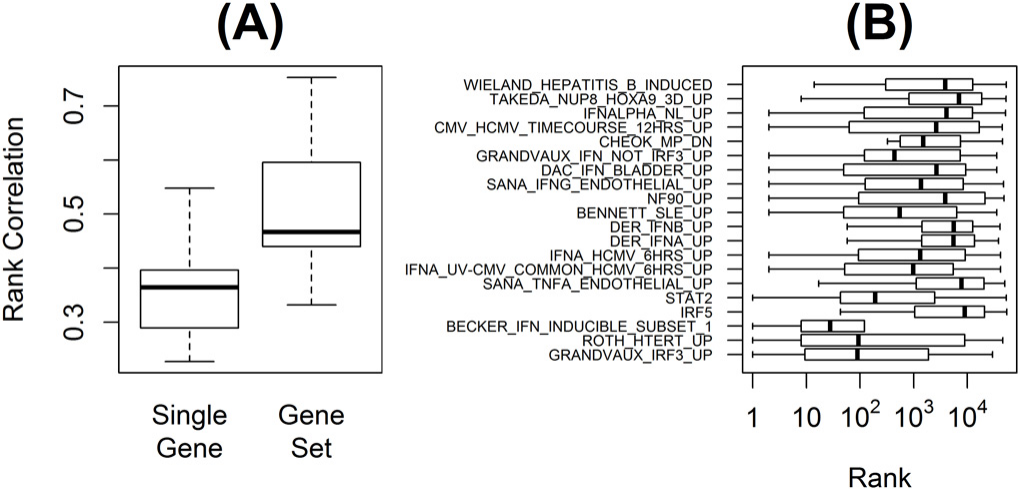
Comparison of single gene and gene set features. **(A)** Shown are the rank correlation coefficients of single gene and gene set feature scores between two exclusive subsets of the seven benchmark data sets. For each data set, its training and test sets were used as exclusive subsets. **(B)** The top 20 predictive gene sets in the trauma benchmark data set are presented. Prediction powers were measured by feature scores. More predictive single genes have lower rank values. Gene sets are noted on the y-axis, and the distributions of their member genes' ranks are plotted along x-axis in a log scale. For each gene set, the left-end of its boxplot represents the highest rank of its member single genes, the right-end represents the lowest rank, and the bar in the middle represents the median rank.

The robustness of features of gene sets and single genes was evaluated by the correlations of the feature scores independently calculated over two exclusively separated subsets of data. For each of the benchmark data set, its training and test sets were used as exclusive subsets. Here, a feature score was calculated with a Cox score model between the expression indices and patient survival times [8]. Rank correlation tests over the benchmark data sets showed that gene set features had higher correlations than single gene features (two-sided paired t-test p-value = 0.001) (Fig 1A). This suggests that informative gene set features in a training set are more likely to be also informative in a test set compared with single gene features.

Moreover, gene sets can efficiently utilize signals of single genes. A summarized gene set signature often represents a higher-level biological signature such as transcriptional regulatory activity [23,24]. This higher-level molecular signature sometimes has a better correlation with patient outcomes than single gene signatures. In the trauma benchmark data set, for example, gene sets such as IRF5, DER_IFNA_UP, DER_IFNB_UP, and CHEOK_MP_DN had strong predictive power while each of their member genes was less informative (Fig 1B). The gene set CHEOK_MP_DN was ranked as one of the top 20 gene sets. However, none of its member genes ranked among the top 100 genes in terms of the single gene feature score. This implies that, while individual genes might not be predictive, the gene set can potentially have higher predictive power.

### The sub-optimal choice of gene set collections

**Fig 2:**
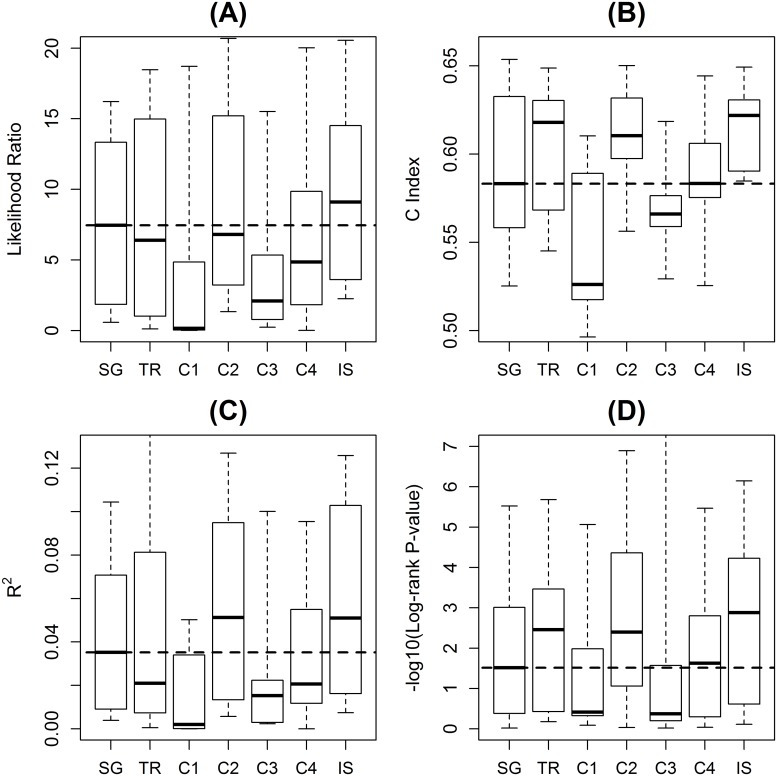
Prediction performance of various gene set collections and single genes. Shown are the prediction performance with TR, C1-4, and IS gene set collections as well as with only single genes (SG). Subplots are for **(A)** likelihood ratio of Cox proportional hazard model fitting, **(B)** Harrell’s C index, **(C)** R^2^, and **(D)** the log-rank test p-value when stratified in the median. Dashed lines represent the median statistics of single gene predictions.

Different collections of gene sets represent different aspects of biology, and their prediction power is expected to vary in different studies. However, we can estimate the overall performance of each collection by studying multiple benchmark data sets. The five collections of gene sets and one integrated collection (Table 1) were tested over each of the benchmark data sets (Table 2). For each data set, a predictor was built with censored survival or recovery time of the training samples, and the survival risks of test samples were predicted. Fig 2 shows the performance of predictions with only gene sets measured by various statistics [20–22].

**Table 1:** Gene set collections used in the study.

Gene set collection	Description	Num. ofgene sets	Num. of unique genes
TR	Transcriptional regulation (TR) gene sets	996	4,955
C1	MSigDB, positional gene sets	350	32,354
C2	MSigDB, curated gene sets	1,890	17,464
C3	MSigDB, motif gene sets	877	15,705
C4	MSigDB, computational gene sets	883	10,083
IS	Integrated super set (IS) of the above five gene set collections	4,956	39,282

**Table 2:** Benchmark data sets used in the study.

Data set	# samples	Disease	Predicted outcomes	Ref.
Trauma	147	Blunt trauma	Recovery	[25]
GSE9782	248	Multiple myeloma	Overall survival	[28]
GSE2658	559	Multiple myeloma	Overall survival	[29,30]
GSE4475	159	Diffuse large B cell lymphoma	Overall survival	[31]
GSE10846	414	Diffuse large B cell lymphoma	Overall survival	[32]
BC RFS	954	Breast cancer	Relapse free survival	[33]
BC DMFS	502	Breast cancer	Distance metastasis free survival	[34]

Gene set collections that are known to be more relevant to the targeted disease showed better performance. The results showed that TR (transcriptional regulation) and C2 (collection 2 of MSigDB[10]) collections had better or similar performance compared with other gene set collections. Biological functions represented by TR and C2 gene sets are related to many diseases including trauma and cancers in the benchmark data sets. For example, transcriptional factors like interferon regulator factors (IRFs) from TR collection as well as signaling pathways like B-cell activation from C2 collection are related to immune responses, which are essential in trauma injury [25]. In contrast, C1 (collection 1 of MSigDB[10]) collection showed poor performance overall. C1 gene sets target large-scale structural variations by grouping about 100 genes on average according to their chromosome positions. Besides, it is worth to note that in some of the data sets using certain collections of gene sets had worse performance than using single genes. It is expected because a particular collection of gene sets only reflects one aspect of the known biology. For example, the collection of C1 gene sets does not include important information on biological functions and signaling pathways. Therefore, it is expected that using C1 collection alone would show worse performance than using single genes. The selection of appropriate collections of gene sets is important for prediction.

Another interesting observation is that high quality gene sets had better predictive power. Gene sets for a biological context inevitably have false positive information. Gene sets with fewer false positives can be considered to have higher quality and confidence. For example, transcriptional regulatory gene sets manually collected by human experts from the primary literature are expected to have higher quality than ones collected by machines through natural language processing [24]. The TR gene sets were of high confidence because they were collected by human experts from the literature. The manually curated functional gene sets of C2 were also of high quality. In contrast, C4 (collection 4 of MSigDB[10]) collection was purely from computational analysis of high throughput data on cancer [9], and C3 (collection 3 of MSigDB[10]) collection was from the inferred binding sites based on sequence motifs [26]. C4 collection of cancer gene sets did not have as good performance as either TR or C2 collections, even though six of the seven benchmark data sets were cancer data sets. In addition, TR collection, which was based on the literature, had better than or at least similar performance with that of the C3 collection, although both contained similar transcriptional regulatory interactions.

While high relevance to the disease and high quality of gene sets can be good criteria when choosing a collection of gene sets for prediction, often none of the existing gene set collections is ideal for a particular disease of the study. Fig 2 showed that there was no additional gain in performance if inappropriate gene set collections were used for prediction. Alternatively, an integrated super (IS) collection of all gene sets can potentially be a sub-optimal choice for many prediction problems. As shown in Fig 2, the IS collection showed reasonably good performance compared with other choices of gene set collections. Moreover, it had better or at least similar prediction performance compared with predictors using single genes alone, even though it also included poorly performed gene set collections. From a mixture of informative and non-informative gene sets, a prediction method tended to select informative gene sets preferentially through the feature selection step. For example, among the top 20 most informative gene sets selected from the IS collection in the trauma data set, 18 gene sets were from C2 collection and two gene sets were from TR collection. This approach of including gene sets from all the different collections for feature selection can potentially be applicable to disease prediction problems in general.

### Hybrid use of gene sets and single genes

Existing collections of gene sets represent only limited biological mechanisms because of our incomplete knowledge. Useful information of single genes may be missed because these genes are not represented properly in the existing gene sets. In this case, a single gene can be included in the features selection as a pseudo gene set with only one member. Here, gene set and single gene features are scored, selected and served as features in the predictor through the same procedure. Since the summarized expression indices of a gene set reflect the mean and variance of its member genes, a single gene can be included directly in the calculation of the feature score and keep its original mean and variance as a gene set.

**Fig 3:**
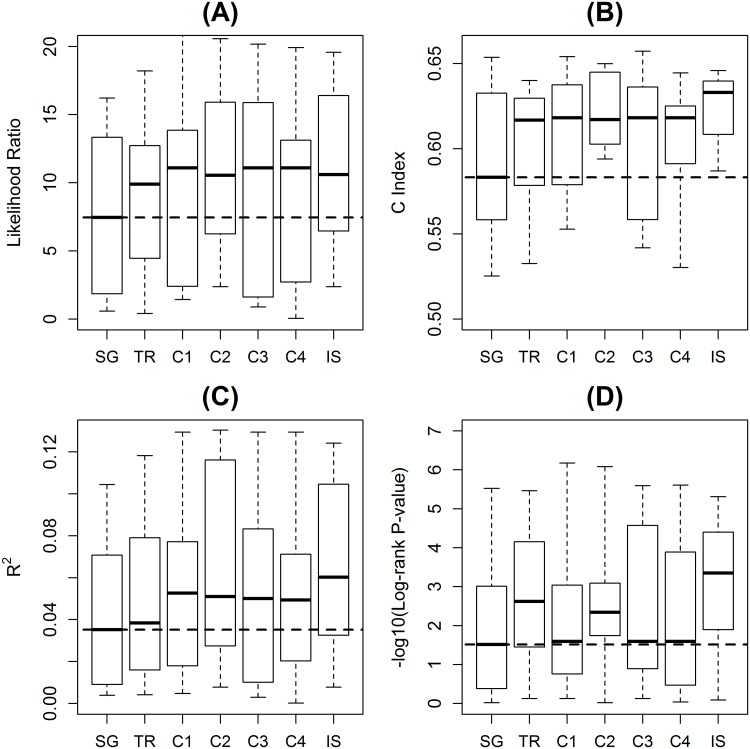
Prediction performance of the proposed hybrid method. Shown are the prediction performance of the proposed hybrid method using both gene sets and single genes for various gene set collections (TR, C1-4, and IS). The prediction performance with only single genes are also shown as a reference (SG). Subplots are for **(A)** likelihood ratio of Cox proportional hazard model fitting, **(B)** Harrell’s C index, **(C)** R^2^, and **(D)** the log-rank test p-value when stratified in the median. Dashed lines represent the median statistics of single gene predictions.

Gene set prediction performance with single genes was evaluated as like in the previous section. As shown in Fig 3, predictions with both gene set and single gene information had better than or at least similar performance compared with predictions with only gene sets but without single gene information in Fig 2. The gene set predictions with and without single genes were directly compared in S2 Fig. The overall performance improvement was tested by paired one-side t-tests in the all 42 prediction cases of the seven benchmark data sets and the six gene set collections. The proposed method of hybrid prediction with both gene sets and single genes showed significant improvements over predictions with only gene sets in log-likelihood ratios (p-value = 1.56×10^-4^), C indices (p-value = 0.022), R^2^ (p-value = 4.68×10^-4^), and log-rank test p-values (p-value = 1.72×10^-4^).

This result implies that information of single genes can potentially help filling in the missing parts of the imperfect knowledge represented by the existing gene sets. As shown in Fig 3, prediction using C1 gene sets only had worse performance than single genes, because the biological context of C1, physical location of genes on the genome, did not reflect the mechanisms of the diseases well. However, when C1 gene sets and single genes were used together, single genes provided information more relevant to the disease, which was missed in the C1 gene sets. For example, in the trauma data set, immune responses were not captured by C1 gene sets while many single genes had functions in immunity. Single genes can partially compensate the missing information of gene sets by providing complementary information that is not included in gene sets.

More importantly, by combining gene set and single gene features, the proposed hybrid method can achieve improved performance over single genes in predictions. On all the seven benchmark data sets, predictions with both of gene set and single gene information had improved performance compared with predictions with single genes alone, or at least showed similar performance. The overall performance improvement was tested by paired one-side t-tests in all of the 42 prediction cases (7 benchmark data sets × 6 gene set collections). The result showed significant improvements of the proposed method over the predictions with only single genes in log-likelihood ratios (p-value = 5.58×10^-5^), C indices (p-value = 6.59×10^-4^), R^2^ (p-value = 2.21×10^-4^), and log-rank test p-values (p-value = 9.47×10^-5^).

### Results of the prediction of patient recovery after trauma as an example

**Fig 4:**
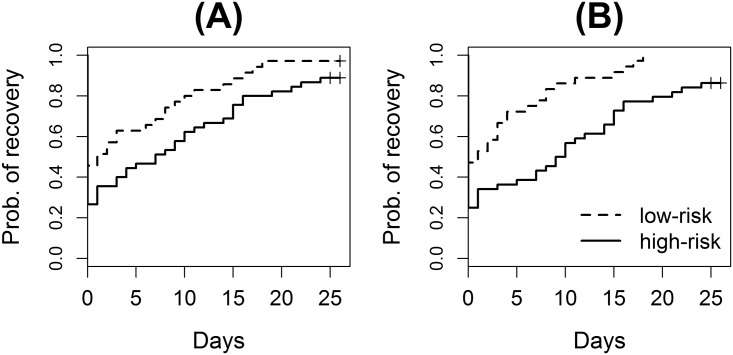
Prediction performance for the trauma benchmark data set. The Kaplan-Meier curves for the recovery of high-risk (solid) and low-risk (dashed) patients according to the recovery risk predicted **(A)** by the conventional method with only single genes and **(B)** by the proposed hybrid method using both single genes and gene sets.

As a detail example, the recovery times of trauma patients were predicted with the IS gene set collection by the proposed hybrid method. Fig 4 shows that the proposed method provided better stratification of trauma patients than a conventional single gene prediction. The low- and high-risk groups stratified from the recovery risks predicted by the gene set method had significantly different recovery outcomes (p-value = 2.23×10^-4^), which was a substantial improvement from the single gene result (p-value = 0.03). P-values were calculated from log-rank tests [20]. Similarly, other metrics were also improved (likelihood ratio: 7.44 to 10.60; R^2^: 0.05 to 0.13; C index: 0.58 to 0.62). In the gene set prediction, the median time to recovery of the low-risk group was 4.2 days, and that of the high-risk group was 9.6 days. Predicted by the single gene method, the low- and high-risk groups had 5.1 and 9.1 days respectively. In addition, all six censored patients, who were not recovered within 24 days from the time of the prediction or died, were classified into the high-risk group in the gene set prediction while the single gene prediction missed two of them. The proposed hybrid method also showed better stratification than the prediction with only gene sets (p-value = 1.31×10^-3^ by a log-rank test). Prediction with only gene sets already achieved an improvement from the result with only single genes because the IS gene set collection provided useful information for the trauma data set. The proposed method further improved the prediction by compensating missing information of the IS gene sets.

From the cross-validations within the training set, the gene set prediction method selected 92 single gene and 30 gene set features to derive a predictor for the trauma data set. The selected gene sets provide useful biological interpretations for the prediction. Nine of the top 20 gene sets (Fig 1B), such as IFNALPHA_NL_UP and DER_IFNB_UP, are sets of genes induced by interferon. These gene sets include many interferon-induced protein (IFI) genes. These gene sets are commonly suppressed in the high-risk group, which might indicate that interferon signaling pathway is less-activated in high-risk trauma patients. In addition, interferon regulator factors (IRFs) are suggested to be important from GRANDVAUX_IRF3_UP. It is also supported by IRF5 gene set that contains genes regulated by IRF5 as well as IRF3 gene set that is one of the selected 30 gene sets but not shown in Fig 1B. STAT2 gene set from TR collection suggests that signal transducer and activator of transcription 2 (STAT2) is activated and induces its target genes. STAT2 can be activated by many cytokines including interferon, which also suggests that the suppression in interferon signaling might be a key biological mechanism related to the delay of patient recovery from trauma injury.

The selected single genes reconfirm the gene set features and fill in missing information of gene sets. Many member genes of the selected gene sets were also involved in the prediction as single gene features. For example, the single gene features of 11 IFIs were also selected as individual features in the prediction. In addition, informative genes missed by gene sets were included as single gene features. As an example, the selected gene sets in the predictor have many guanylate binding protein (GBPs) genes, but these gene sets do not include GBP5 as a member. The prediction algorithm selected GBP5 which provided additional information as a single gene feature.

On the other hand, gene sets also include additional genes that are not included in the predictor as single genes. The selected 92 single gene features include three human leukocyte antigen (HLA) genes, HLA-DMA, HLA-DMB, and HLA-DRB1. Many other HLAs are not included in the selected single genes because they are less informative in the training set even though they are essential factors in immune response. In contrast, the selected gene sets in the trauma prediction have more HLA genes—HLA-A/B/C/E, DRA, DPA and DQA1 missed by single genes as well as HLAs already included in the single gene features—because they are grouped according to their similar biological functions. Here the gene set approach therefore utilizes the information from these additional HLA genes to extract stronger signature of trauma injury.

## Conclusion

We studied a hybrid approach using both single genes and gene sets in the prediction of patient outcomes by investigating three major issues: incorporation of gene set information, selection of gene set collections, and compensating the incomplete knowledge represented by gene sets. First, in order to utilize gene set information in a prediction problem, we propose to summarize gene set features from expression levels of multiple single genes. The gene set features were shown to have robust information by summarizing the weaker signatures of its member genes. Second, the use of an integrated super (IS) collection of multiple gene set collections showed similar or better performance compared with the use of individual gene set collections, suggesting that the feature selection procedure can successfully select informative gene sets from all the gene sets included in the analysis. Third, the incomplete knowledge of biology and diseases represented by the collections of gene sets could be partially compensated by including single gene features. This hybrid approach was tested over the seven benchmark data sets. Compared with single gene predictions, the proposed method was able to achieve improved prediction for survival risk. Compared with predictions with only gene sets, the hybrid method showed robust performance regardless of gene set collections used in the prediction. The development of utilizing biological knowledge is expected to be applicable in a wide range of prediction problems in clinical genomics and personalized medicine.

For the successful use of previously accumulated knowledge in the analysis of genomic data, which is often referred as knowledge-based analysis, our knowledge itself is the most essential factor. It encourages for our research community to build good knowledge bases. The performance comparison of gene sets in this work confirms important characteristics of good knowledge bases studied before. Knowledge bases with comprehensive, high quality and direct information show better performance in the prediction of transcriptional regulatory relations in yeast [24]. Gene sets, which are a kind of knowledge bases, show similar characteristics in patient outcome predictions. Gene sets relevant to a disease have better prediction power, which corresponds to the directness of knowledge. In addition, gene sets from literatures are superior to those from computational inference, which corresponds to high-quality of knowledge. These characteristics of good knowledge bases guide us to establish better knowledge bases or gene sets for the future knowledge-based analysis.

## Materials and Methods

### Collections of gene sets utilized in this study

We identified the collections of available gene sets for the study. Table 1 lists five collections of gene sets and one integrated super collection as well as the numbers of gene sets and unique genes in each collection. The Molecular Signature Database (MSigDB) provided gene sets categorized by chromosome positions (C1), biological functionality (C2), cis-regulatory motifs (C3), and gene modules computationally inferred from cancer data sets (C4) [10]. In addition, a collection of transcriptional regulation (TR) gene sets, each of which includes a set of target genes regulated by a transcriptional factor, was defined from commercial Ingenuity database [12] and Pathway Studio database [27]. Note that the TR gene sets were curated from the literature while C3 gene sets were computationally inferred from the regulatory motifs of transcriptional factors. Finally, we compiled an integrated super (IS) collection of all the five collected gene sets. The IS collection consists of 4,956 gene sets, each of which has about 100 member genes on average.

These gene sets provide useful prior knowledge on biological mechanisms and diseases. For examples, transcriptional regulatory networks are represented in C3 and TR gene sets, and metabolic and signaling transduction pathways are represented in C2. C2 gene sets also represent the gene expression patterns measured by high-throughput experiments under various conditions. C1 gene sets group genes by cytogenetic bands, which would be useful to detect large scale genomic variations. C4 sets represent modules identified from cancer studies.

### Benchmark data sets

The performance of the proposed gene set prediction method was evaluated over several publically available data sets. Seven data sets of large patient populations of different diseases were chosen as benchmark data sets (Table 2). For each data set, independent training and test sets were designated as noted in the original paper. A trauma data set was collected from n = 147 severe blunt trauma patients at day 4 after injury [25], and patient recovery risks from trauma injury was predicted. Patients who died or were not recovered within 24 days from the time of the prediction were censored. Training and test sets were divided according to chronicle cohorts. Two multiple myeloma data sets of GSE9782 (n = 248) [28] and GSE2658 (n = 559) [29,30] were tested for predictions of overall survival risks. Two lymphoma data sets of GSE4475 (n = 158) [31] and GSE10846 (n = 414) [32] were analyzed for the prediction of overall survival risks. Finally, for breast cancer, two collections of public data sets, BC RFS (n = 954) [33] and BC DMFS (n = 502) [34], were tested. For the prediction of relapse free survival (RFS) risks, the expression profiles collected and processed by Acharya, *et al*. [33] were used. In this data set, the collection of GSE7849, 3143, 2034, and 4922 was served as a training set, and GSE6532 was used as a test set. For the prediction of distant metastasis free survival (DMFS) risks, 502 patient samples collected from three public data sets were tested according to Schmidt, *et al*. [34]. In this data set, GSE11121 was used as a training set, and the combined set of GSE6532 and GSE7390 as a test set.

The data sets of trauma, multiple myeloma and lymphoma were each from a single study. For these data sets, all the samples were profiled by the same array platform and protocol, and no further adjustment was performed. The data sets of BC, which include BC RFS and BC DMFS, were meta-data from multiple studies. In these sets, samples were processed through different protocols by different study groups, and we therefore performed additional standardization. For the BC RFS data set, we obtained the pre-standardized expression matrix from the authors of the original paper [33], which was adjusted by a cross-platform standardization algorithm, ComBat [35]. For the BC DMFS data set, we standardized gene expression within each data set so that each data set had the same means and variances with the overall means and variances. Therefore, individual genes of these meta data have comparable means and variances across data sets.

For the meta-analysis of the breast cancer data sets, the selection and use of the sub data sets follow the settings of the original research papers [33,34]. The detail patient characteristics, including age, grade, tumor size, estrogen receptor status and lymph node status, are presented in the original papers. There is no significant difference in the patient characteristics among the data sets used for training and test sets in the predictions of this work.

### The overall prediction procedure

The overall flow of the prediction in this work is described in S1 Fig. Starting from a single gene expression matrix of a training set, the proposed method first calculates the gene set expression using the predefined gene sets. This gene set expression is handled like single gene expression. The gene set and single gene expression indices are served as prediction features. For each of gene sets and single genes, its feature score is calculated based on the correlation of its expression and the survival outcome. Through the cross-validation within the training set, top features with the largest scores are selected as the final prediction features. These final features are fed into Semi-Supervised Principal Component (SuperPC) method [8] and used to build the final predictor. The final predictor is applied to a test set that is independent and exclusive with the training set. The performance of the prediction is measured from the test set. The R code for the proposed method was deposited in GitHub (jseok79/HybridPred).

### Calculation of expression indices of gene sets from gene expression profiles

The expression index of a gene set is calculated by summarizing those of its member single genes. Let *e*_*ij*_ be an expression value of single gene *i* for sample *j*. *e*_*ij*_, single gene expression, is standardized so that it has 0 mean and 1 standard deviation. Let *e'*_*ij*_ be the standardized expression value of *e*_*ij*_. The expression index of a gene set is calculated by summarizing over the standardized expression value of the individual genes which belong to this gene set using maxmean statistic [14], as describe below two steps.

Step 1. For gene set *g* with *N*_*g*_ members of single genes, its expression index in sample *j*, *u*_*gj*_, is calculated as following:

$$u_{gj}=\text{abs}\max\left[\frac{1}{N_{g}}\sum_{i\in GS_{g}}{\left({e^{\prime }}_{ij}\right)}_{+},\frac{1}{N_{g}}\sum_{i\in GS_{g}}{\left({e^{\prime }}_{ij}\right)}_{-}\right]$$

where *GS*_*g*_ denotes the set of member genes of gene set *g*. The cleavage and absmax functions are defined as following:

$${\left(x\right)}_{+}=\left\{ \begin{array}{l} x,\;x\geq 0\\ 0,\;x<0 \end{array}\right.\;,\;{\left(x\right)}_{-}=\left\{ \begin{array}{l} 0,\;x\geq 0\\ x,\;x<0 \end{array}\right.\;,\text{ absmax}\left[\text{x,y}\right]=\left\{ \begin{array}{l} x,\;\left|x\right|\geq \left|y\right|\\ y,\;\left|x\right|<\left|y\right| \end{array}\right.$$

Step 2. the summarized *u*_*gj*_ is then standardized to mean 0 and standard deviation 1, and scaled again to reflect the means and standard deviations of its member genes. Let *u'*_*gj*_ be the standardized value of *u*_*gj*_. The scaled gene set expression *x*_*gj*_ is given as following:

$$x_{gj}={u^{\prime }}_{gj}\sqrt{\frac{\sum_{i\in GS_{g}}\sigma_{i}^{2}}{N_{g}}}+\frac{\sum_{i\in GS_{g}}\mu_{i}}{N_{g}}$$

where *μ*_*i*_ and *σ*_*i*_ denote the mean and standard deviation of expression level *e*_*ij*_'s across samples. The final expression index of a gene set has the averaged mean and variance of the expression values of its member genes. This also makes it straightforward to integrate the expression indices of gene sets with the expression indices of additional single genes.

### Training a predictor using gene set and single gene features

The calculated gene set expression indices can be straightforwardly incorporated as prediction features in SuperPC method which was originally developed using single gene expression indices [8]. In SuperPC, the feature score of each gene set and single gene is calculated by a Cox score, as the measure of the correlation between the expression index and patient outcome. More precisely, the Cox score measures the fitness of the expression indices with respect to the censored survival times in a well-known Cox proportional hazard (CoxPH) model [21]. The CoxPH model and Cox scores have been widely used to infer significantly associated genes with survival outcomes in gene expression analysis [6–8]. The Cox score can be considered as a good measurement for the predictive power. Only informative features selected according to feature scores are fed into the principal component analysis. The R code provided by SuperPC was used in the calculation of the Cox scores.

Since the feature score of a gene set which was summarized over a number of its member genes is likely to have more robust information than that of a single gene [15], a weighted feature matrix was used to derive the predictor. Let feature *x*_*1*_, *x*_*2*_, …, and *x*_*n*_ be selected as informative ones. Then, a predictor is calculated from the first a few principal components of the following weighted feature matrix:

$$\left[\begin{array}{cccc} \sqrt{N_{1}} & 0 & \cdots & 0\\ 0 & \sqrt{N_{2}} & & 0\\ \vdots & & \ddots & \vdots \\ 0 & 0 & \cdots & \sqrt{N_{n}} \end{array}\right]\left[\begin{array}{c} x_{1}^{T}\\ x_{2}^{T}\\ \vdots \\ x_{n}^{T} \end{array}\right]$$

where *N*_*i*_ denotes the number of member gene of feature *i*. If feature *i* is from a single gene, *N*_*i*_ becomes 1. This is a generalized principal component analysis for gene sets. If all features are from single genes, it is identical to the conventional principal component analysis. The weights emphasize gene set features summarized from more member genes.

### Prediction of patient survival risk

For given training and test sets, a predictor for patient risks was built from the training set by fitting the censored survival or recovery times with Cox proportional hazard models, and it was applied to predict the survival or recovery risks of patients in the exclusive test set. The predictor was derived without referring to any information of test samples. For example, the mean and standard deviation of each single gene which are required for the calculation of expression indices of the gene sets were estimated only from training samples.

First, informative features were selected from cross-validations. Briefly, the feature scores of gene set and single gene features were calculated from a training set, and only features of which scores were higher than a threshold were selected for the further analysis. The threshold was obtained from cross-validations within a training set. A training set was randomly divided into three groups. Predictors were derived from the two groups with various thresholds and applied to the third group. The performance of the prediction at each threshold value was evaluated. The threshold value with the best performance in the cross-validation was chosen for the feature selection. The cross-validation was repeated 100 times to obtain a robust threshold.

Once features were selected from a training set, a predictor was calculated by principal components of the weighted feature matrix (see the above section) of the training set. Here, for simplicity the first one principal component of the feature matrix was used as the predictor while it is possible to use multiple principal components together. The risk score of each sample was calculated as the projection of each sample expression profile to the predictor.

## Supporting Information

S1 Fig A diagram of the flow for the proposed prediction method. (PNG)

S2 Fig Summarized performance comparison of the proposed prediction method. Shown are the prediction performance with TR, C1-4, and IS gene set collections as well as only single genes (SG). The proposed hybrid predictions (GS+SG; red) and prediction with only gene sets (GS only; green) are shown. Subplots are for **(A)** likelihood ratio of Cox proportional hazard model fitting, **(B)** Harrell’s C index, **(C)** R^2^, and **(D)** the log-rank test p-value when stratified in the median. Dashed lines represent the median statistics of single gene predictions.
(TIFF)
