# Supplementary figures and images for: A Hybrid Approach of Gene Sets and Single Genes for the Prediction of Survival Risks with Gene Expression Data
Source: PLoS One. 2015 May 1;10(5):e0122103. doi: 10.1371/journal.pone.0122103 (PMC4416884; doi:10.1371/journal.pone.0122103)

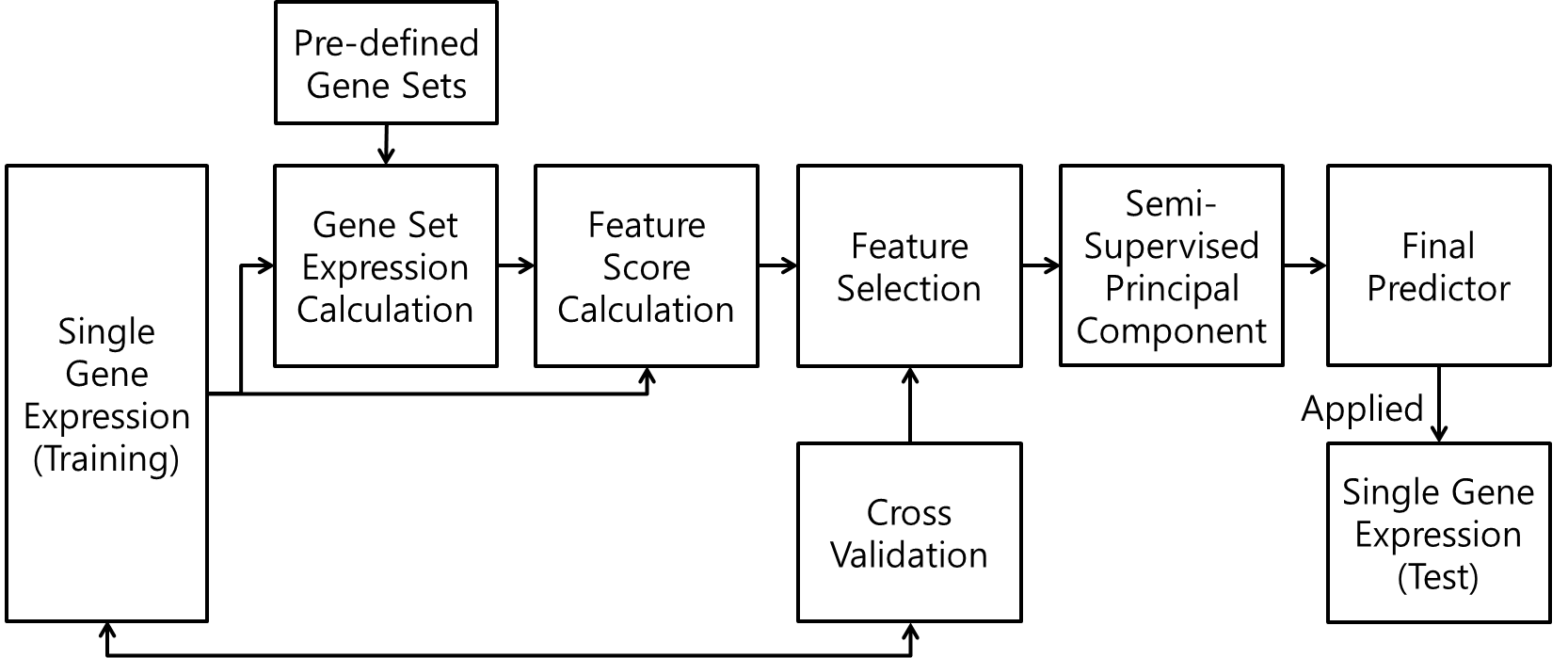

Supplement: S1 Fig — (PNG) [file pone.0122103.s001.png]

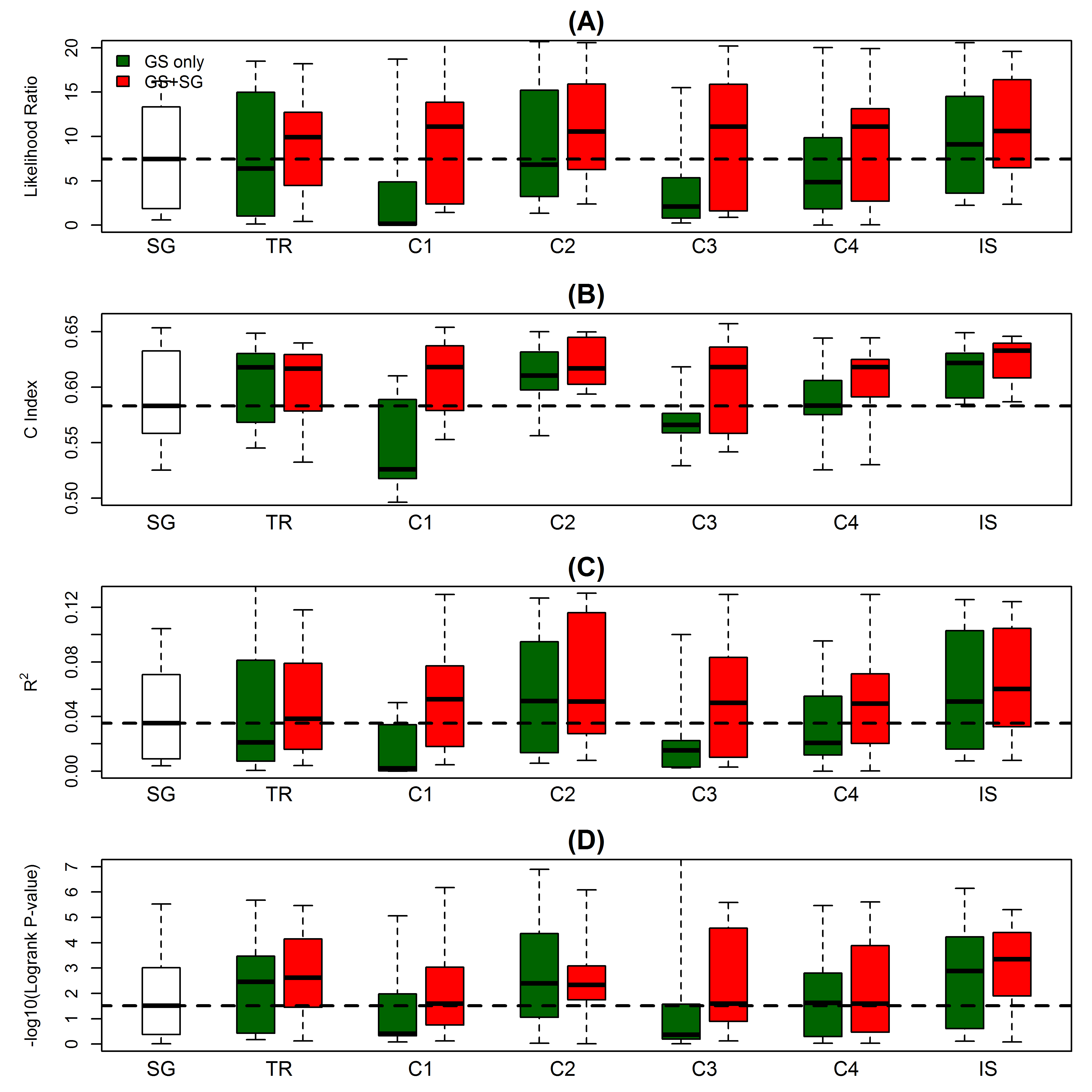

Supplement: S2 Fig — Shown are the prediction performance with TR, C1-4, and IS gene set collections as well as only single genes (SG). The proposed hybrid predictions (GS+SG; red) and prediction with only gene sets (GS only; green) are shown. Subplots are for (A) likelihood ratio of Cox proportional hazard model fitting, (B) Harrell’s C index, (C) R2, and (D) the log-rank test p-value when stratified in the median. Dashed lines represent the median statistics of single gene predictions. (TIFF) [file pone.0122103.s002.tiff]
